# The Peroxisome-Mitochondria Connection: How and Why?

**DOI:** 10.3390/ijms18061126

**Published:** 2017-05-24

**Authors:** Marc Fransen, Celien Lismont, Paul Walton

**Affiliations:** 1 Laboratory of Lipid Biochemistry and Protein Interactions, Department of Cellular and Molecular Medicine, KU Leuven, University of Leuven, 3000 Leuven, Belgium; celien.lismont@kuleuven.be; 2 Department of Anatomy and Cell Biology, University of Western Ontario, London, ON N6A 3K7, Canada; pwalton@uwo.ca

**Keywords:** peroxisomes, mitochondria, fatty acid oxidation, reactive oxygen species, interorganelle crosstalk, organelle abundance, organelle dysfunction, human disease

## Abstract

Over the past decades, peroxisomes have emerged as key regulators in overall cellular lipid and reactive oxygen species metabolism. In mammals, these organelles have also been recognized as important hubs in redox-, lipid-, inflammatory-, and innate immune-signaling networks. To exert these activities, peroxisomes must interact both functionally and physically with other cell organelles. This review provides a comprehensive look of what is currently known about the interconnectivity between peroxisomes and mitochondria within mammalian cells. We first outline how peroxisomal and mitochondrial abundance are controlled by common sets of *cis*- and *trans*-acting factors. Next, we discuss how peroxisomes and mitochondria may communicate with each other at the molecular level. In addition, we reflect on how these organelles cooperate in various metabolic and signaling pathways. Finally, we address why peroxisomes and mitochondria have to maintain a healthy relationship and why defects in one organelle may cause dysfunction in the other. Gaining a better insight into these issues is pivotal to understanding how these organelles function in their environment, both in health and disease.

## 1. Introduction

Eukaryotic cells are endowed with a diverse array of structurally and functionally discrete membrane-enclosed organelles. Such compartmentalization provides several distinct benefits, including (i) the creation of different local environments that facilitate specific metabolic functions; (ii) the sequestering of reaction intermediates and potentially toxic metabolites; and (iii) the ability to perform specific functions without interfering with other cellular processes [1]. However, cell organelles are not isolated entities and in order to exert their activities, they must actively communicate and interact with other subcellular compartments. This review provides a comprehensive overview of what is currently known about the functional and physical connections between peroxisomes and mitochondria, with a focus on mammals.

Peroxisomes are remarkably dynamic organelles that adapt their number, morphology, and activity depending on the tissue, organ, and nutritional state [2]. For an in-depth review of these adaptations, the reader is referred to a comprehensive review by Schrader and colleagues [3]. In mammals, peroxisomes play an indispensable role in different biochemical pathways, including ether-phospholipid biosynthesis, fatty acid α- and β-oxidation, bile acid and docosahexaenoic acid synthesis, glyoxylate metabolism, amino acid catabolism, polyamine oxidation, and the metabolism of reactive oxygen and nitrogen species (ROS and RNS, respectively) [4,5,6,7,8]. In addition, during the last decade, it has become clear that mammalian peroxisomes are not solely metabolic organelles but can also serve as signaling platforms that modulate diverse physiological and pathological processes including inflammation, innate immunity, and cell fate transitions [9]. Abnormalities in any of these metabolic or signaling functions can be directly or indirectly linked to a growing number of diseases, ranging from rare genetic disorders (e.g., Zellweger syndrome, X-linked adrenoleukodystrophy, and acatalasemia) [8] to more common age-related disorders such as diabetes, neurodegenerative disease, and cancer [10,11].

Like peroxisomes, mitochondria are also dynamic organelles that continuously adapt their number, morphology, and function to prevailing environmental conditions [12]. In mammals, these organelles play a central role in many metabolic processes including–among others–adenosine triphosphate (ATP) generation, β-oxidation of fatty acids, ketone body production, and iron-sulfur cluster synthesis [13,14]. Mitochondria also operate as important platforms in cellular signaling networks that impact on a wide array of biological processes, ranging from gene expression and immune responses to cell differentiation and cell death [15]. As such, it is not surprising that mitochondrial dysfunction has been proposed to contribute to the pathogenesis of numerous metabolic (e.g., metabolic syndrome and obesity) and neurodegenerative (e.g., Parkinson’s and Alzheimer’s disease) disorders [13].

In the following sections, we first focus on the cellular and molecular processes underlying co-regulation of peroxisomal and mitochondrial abundance (see Section 2). Next, we discuss the mechanisms potentially involved in peroxisome-mitochondrial communication (see Section 3). We also outline how these organelles cooperate in various metabolic (see Section 4) and signaling (see Section 5) pathways. Finally, we provide an overview of what is currently known about the interplay between peroxisomes and mitochondria under various disease conditions (see Section 6).

## 2. Control of Peroxisomal and Mitochondrial Abundance

**Figure 1:**
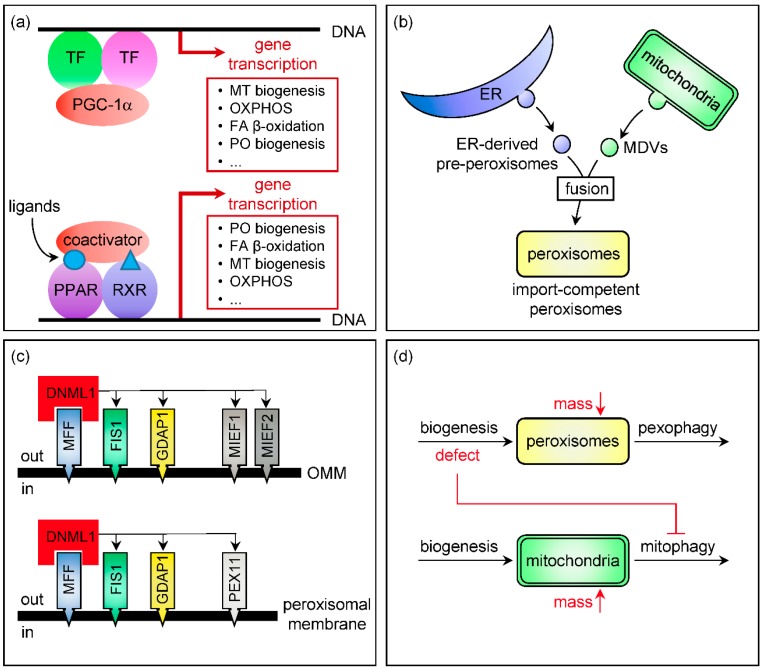
Peroxisomal and mitochondrial abundance are controlled by common sets of *cis*- and *trans*-acting factors. (**a**) Transcriptional control of peroxisome and mitochondrial biogenesis; (**b**) De novo peroxisome biogenesis requires mitochondria-derived vesicles; (**c**) Peroxisomes and mitochondria share components of their fission machinery. Shared and organelle-specific components of the fission machinery are indicated in color and gray, respectively; (**d**) Defects in peroxisome biogenesis impinge on mitophagy. Decreases and increases in organellar mass are indicated by red downright and upright arrows, respectively. Black horizontal arrows represent the formation of new organelles (left arrows) or the removal of damaged/dysfunctional organelles (right arrows). The red T-bar arrow denotes inhibition. ER, endoplasmic reticulum; FA, fatty acid; FIS1, mitochondrial fission protein; GDAP1, ganglioside-induced differentiation-associated protein; MDVs, mitochondria-derived vesicles; MFF, mitochondrial fission factor; MT, mitochondria/mitochondrial; OMM, outer mitochondrial membrane; OXPHOS, oxidative phosphorylation; PEX11, peroxin 11; PO, peroxisome/peroxisomal; TF, transcription factor.

Fluctuations in organelle abundance can be expected to have significant effects on their functional output, and—to adjust organelle quantity in response to changing environmental and developmental stimuli—cells are equipped with mechanisms that coordinate the formation of new organelles and their subsequent degradation once they are excessive or non-functional (Figure 1). In mammals, new peroxisomes are formed de novo (e.g., from the ER [16,17] or by fusion of mitochondria-derived vesicles and ER-derived pre-peroxisomes (Figure 1b) [18]) or by asymmetric growth and division of pre-existing organelles [19,20]. Damaged or superfluous peroxisomes are mainly degraded by the autophagy-lysosome pathway, in a process known as pexophagy [21,22]. Mitochondria are highly dynamic reticular organelles whose number and morphology is continuously remodeled by fission and fusion [23]. Redundant or dysfunctional mitochondria are selectively removed by autophagy through a process called mitophagy [24]. Unlike mitochondria, mature peroxisomes cannot fuse with one another [19,25]. In the following sections, we further discuss the mechanisms underlying co-regulation of peroxisomal and mitochondrial abundance and activity in mammalian cells.

### 2.1. Transcriptional Control of Organelle Biogenesis

As discussed below, multiple cellular processes (e.g., fatty acid β-oxidation (see Section 4.1) and antiviral innate immune signaling (see Section 5.2)) require synchronized action of both peroxisomes and mitochondria. Over the past decades, it has become increasingly apparent that both organelles share transcriptional regulatory mechanisms that coordinate their abundance and enzyme content (Figure 1a) [26]. The expression of many genes involved in peroxisomal fatty acid β-oxidation and proliferation is controlled by transcription factors of the PPAR family [27,28,29]. PPARs (PPARα, PPARγ, and PPARδ) are ligand-activated nuclear receptors that act in concert with retinoid X receptors (RXRs) to regulate a variety of physiological processes (e.g., lipid and carbohydrate metabolism, cellular differentiation, and tumorigenesis) [28,30], and natural and synthetic PPAR agonists include dietary lipids and their metabolites, fibrates, and thiazolidines [31]. Each PPAR subtype displays a distinct tissue expression pattern and substrate specificity and regulates the expression of different target genes [32]. In addition, PPAR activity can be modulated by a number of coactivator (e.g., PGC-1α) and corepressor (e.g., NCOR) proteins [33]. Although best known for their role in peroxisome proliferation, PPARs have also been reported to modulate the expression of genes involved in mitochondrial β-oxidation (e.g., through PPARα in mouse liver [34] or PPARβ in murine adipocytes and cardiomyocytes [35]) and biogenesis (e.g., through PPARα in monkey liver [36] and PPARγ in differentiated human neuroblastoma cells [37]).

Currently, it is well established that PGC-1α, a transcriptional coactivator, functions as a master driver of mitochondrial biogenesis and function [38]. PGC-1α cooperates with a variety of other nuclear receptors (e.g., PPARs and NRFs) to promote enhanced expression of genes encoding mitochondrial biogenesis factors, oxidative phosphorylation subunits, and antioxidant enzymes [39]. Over the past decade, emerging evidence has shown that PGC-1α also functions as a positive regulator of peroxisome biogenesis in various tissues, including liver [40], brown fat [40], and skeletal muscle [41]. Interestingly, this process apparently does not require PPARα, at least not in those tissues in which the PPARα-PGC-1α relationship was examined [40]. Taken together, these findings support the concept that peroxisomal and mitochondrial abundance and activity are co-regulated at the transcriptional level in a PPAR- and PGC-1α-dependent manner.

### 2.2. Organelle Multiplication by Fission

Peroxisomes and mitochondria can proliferate by fission of pre-existing organelles [42,43,44]. A major breakthrough in the field was the discovery that these organelles share multiple components of their fission machinery (e.g., mitochondrial fission protein (FIS) 1 [45], mitochondrial fission factor (MFF) [46], ganglioside-induced differentiation-associated protein (GDAP) 1 [47], and dynamin 1-like protein (DNM1L) [48,49]) (Figure 1c). DNM1L is a predominantly cytosolic large guanosine triphosphatase that, upon recruitment to organellar membranes, can self-oligomerize into helices, thereby catalyzing membrane fission and vesicle release in a GTP hydrolysis-dependent manner [50]. Recruitment of this protein to peroxisomal or mitochondrial division sites depends on membrane adaptor proteins such as FIS1, MFF, and GDAP1 [42,43]. Although overexpression or downregulation of any of these adaptor proteins has been shown to induce peroxisomal and mitochondrial fragmentation or elongation, respectively [45,46,47,51], the precise function of each of these proteins in organelle fission remains to be determined. However, in this context it is interesting to note that MFF appears to function as the major docking protein for DNM1L on the organellar membrane, and that FIS1 functions downstream of Mff [51,52]. It is also not clear why silencing of either of these proteins results in perinuclear clustering of mitochondria, but not peroxisomes [49,53], a phenotype that is also observed upon disruption of DNM1L function [49,53].

To fully understand the complexity of how DNM1L may spatially coordinate peroxisomal and mitochondrial fission, it is important to be aware that this protein can also be recruited to mitochondria through interaction with mitochondria-specific adaptor proteins (e.g., mitochondrial elongation factor (MIEF) 1 and 2), and that overexpression of MIEF1 or MIEF2 causes extensive mitochondrial and peroxisome elongation through a dominant-negative mechanism wherein DNM1L is inactively sequestered at the mitochondrial surface [54]. In addition, the interaction of DNM1L with its adaptor proteins is regulated by several posttranslational modifications (e.g., phosphorylation, ubiquitylation, and sumoylation) [42,44]. Finally, the physiological role of peroxisomal and mitochondrial fission in mammals remains poorly understood. However, as this process appears to be critical for the efficient elimination of dysfunctional organelles and maintenance of cellular health [42,55,56], it may not be surprising that defects in any of the shared components of their fission machinery can result in severe diseases (see Section 6.2).

### 2.3. Organellophagy

Multiple studies have documented that defects in peroxisome biogenesis [57,58,59] or mitochondrial function [60,61] coincide with an increase in the volume of the other subcellular compartment (for details, see Section 4.2.2 and Section 5.1.1). Until recently, it was thought that this phenomenon represented a compensatory mechanism in which the biogenesis of one organelle is upregulated to counteract dysfunction of the other. However, as it was lately shown that a deficiency in peroxin 13 (PEX13), a membrane-bound component of the peroxisomal matrix protein import machinery, induces mitophagy defects in cultured mouse embryonic fibroblasts [62], it is highly plausible that the increase in mitochondrial volume observed in various tissues (e.g., brain [62] and liver [57]) upon peroxisome inactivation is at least partially due to inefficient mitochondrial turnover (Figure 1d). This point of view is strengthened by the observation that, in mouse liver, chronic or acute inactivation of PEX5, the cycling import receptor for peroxisomal matrix proteins, increases mitochondrial volume several-fold despite the fact that the primary driver of mitochondrial biogenesis, PGC-1α, is strongly downregulated at the transcript and protein level [57]. The molecular mechanisms underlying these observations remain to be determined. In addition, it is not yet clear if and how defects in mitochondrial function impinge on pexophagy.

## 3. Peroxisome-Mitochondria Communication

**Figure 2:**
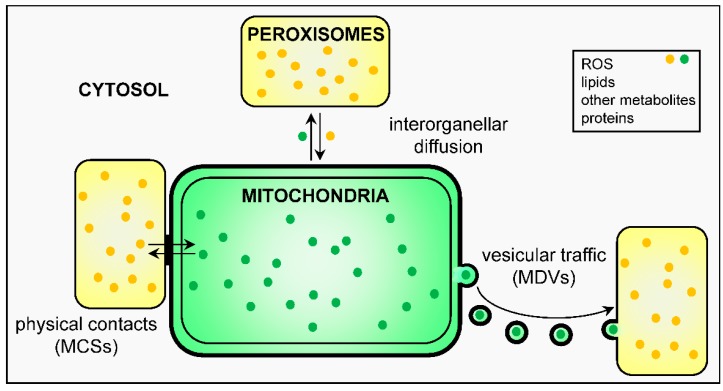
Schematic model of the mechanisms potentially involved in peroxisome-mitochondrial communication. The yellow and green dots represent peroxisome- and mitochondria-derived molecules, respectively. MCS, membrane contact site; MDV, mitochondria-derived vesicle.

To fulfill their functions, cell organelles constantly communicate with their environment. In the next sections, we focus on the mechanisms that peroxisomes and mitochondria employ to stay in touch (Figure 2). For more information on how these organelles exchange information with other subcellular compartments, we refer the reader to another recent review [26].

### 3.1. Physical Contact Sites and Tethers

Over the years, mounting evidence has been collected that peroxisomes and mitochondria can come into close apposition, at least transiently or under certain circumstances. For example, already in the 1970s, a comparative ultrastructural analysis of the myocardium of rodents and primates revealed a close spatial association between these organelles [63]. More recently, additional evidence for a physical association between peroxisomes and mitochondria was obtained by (i) analytical density gradient centrifugation of fractions derived from rat liver [64] and human hepatoma cells [65,66]; (ii) confocal microscopy studies [65,67]; and (iii) proximity ligation assays [67]. Interestingly, the degree of contact can dynamically change depending on the cell type and physiological circumstances. For instance, in human hepatoma cells, the interaction between peroxisomes and mitochondria is strongly increased upon activation of the retinoic acid-inducible gene 1 protein (RIG-I) pathway (see Section 5.2) [65,66]; and in mouse Leydig tumor cells, a similar phenomenon is observed upon treatment of the cells with dibutyryl cyclic adenosine monophosphate (cAMP), a potent signaling molecule for steroidogenesis [67]. At present, one peroxisome-mitochondria tethering complex has been identified in mammals [67]. The core component of this complex is a splice variant of enoyl-CoA δ isomerase 2 (ECI2 isoform A), an acyl-CoA binding domain-containing protein containing a cleavable N-terminal mitochondrial targeting signal as well as a C-terminal peroxisomal targeting signal. Expression of this isoform in Leydig tumor cells (e.g., ectopically or through treatment of the cells with dibutyryl cAMP) has been reported (i) to promote peroxisome-mitochondria apposition, most likely through the simultaneous binding of both targeting signals to the peroxisomal and mitochondrial protein import complexes; and (ii) to increase basal and hormone-stimulated steroid formation, plausibly through favoring the interorganellar exchange of metabolites involved in steroid biosynthesis [67]. In yeast, it has been shown that peroxisomes preferentially localize to specific mitochondrial subdomains such as mitochondria-associated ER membranes (MAMs) and sites of acetyl-CoA synthesis [68], and that these peroxisome-mitochondria contact sites are established by direct interaction between Pex11, a peroxisomal membrane protein, and Mdm34 [69], a component of the ER-mitochondria encounter structure (ERMES) [70].

### 3.2. Vesicular Transport

Organelles in the secretory and endocytic pathways have evolved the capacity to communicate with each other through the release of small vesicles. Approximately a decade ago, the McBride lab discovered that vesicles are also a common form of communication for mitochondria [71], and that a subgroup of mitochondria-derived vesicles (MDVs) transports cargo from these organelles to a subpopulation of peroxisomes [72]. These MDVs, which are formed independently of DNM1L and enriched for the mitochondrial E3 ubiquitin protein ligase (MUL) 1, are transported from mitochondria to peroxisomes in a retromer-dependent manner (retromer is a membrane-associated coat complex essential for endosome-to-Golgi retrograde transport) [73]. The physiological role of these MUL1-positive MDVs has been the subject of debate [74]. However, very recently it has been demonstrated that, in human patient fibroblasts lacking peroxisomes, MUL1-positive MDVs fuse with ER-derived pre-peroxisomes to form vesicular structures that subsequently mature into fully functional peroxisomes [18]. Whether or not these MDVs serve a similar function in wild-type cells, remains to be determined. In addition, no data currently exist for the existence of a vesicular transport pathway from peroxisomes to mitochondria.

### 3.3. Biological Messengers

As discussed in detail in other sections below, peroxisomes and mitochondria have the ability to convey information to each other through the release of biological messengers such as ROS (see Section 5.1.1), lipids (see Section 5.1.2), or other metabolites (see Section 5.1.3). A key step in this process is the transport of these molecules across the organellar membranes. Depending on the type of molecule and membrane involved, potential mechanisms may include passive membrane permeation (e.g., through pores or channels) or active influx or efflux (e.g., by specific transporters). In the following paragraphs, we briefly compare the mechanisms of solute transport across the peroxisomal and mitochondrial membranes. For more detailed information, we refer the reader to other reviews.

Peroxisomes and mitochondria are surrounded by one and two membranes, respectively, each displaying different permeability properties: the OMM contains porin proteins that form transmembrane channels large enough to allow free passage of molecules up to 5 kDa [75]; the inner mitochondrial membrane (IMM) is impermeable to inorganic ions and water-soluble metabolites, and transport of these compounds across the IMM requires the presence of a large set of transporters [76]. The peroxisomal membrane contains non-selective channels (e.g., peroxisomal membrane protein 2 (PXMP2) in mammals [77]) that allow free transmembrane movement of solutes with molecular masses up to 300–400 Da but prevent diffusion of larger molecules [78]. Transmembrane transfer of larger molecules (e.g., fatty acids, acetyl-CoA, and ATP) across the peroxisomal membrane requires specific transporter proteins. Examples of transporters that have been identified include (i) three members (ABCD1-3) of the ATP-binding cassette half-transporters of subfamily D (ABCD), which catalyze the transmembrane transport of the substrates for peroxisomal fatty acid oxidation [79]; (ii) the adenine nucleotide transporter SLC25A17, which functions as a transporter of CoA, flavin adenine dinucleotide (FAD), flavin mononucleotide, and adenosine monophosphate, and—to a lesser extent—of nicotinamide adenine dinucleotide (NAD^+^) and adenine dinucleotide phosphate (ADP) [80,81]; and (iii) three members (SLC16A1, SLC16A4, and SLC16A7) of the monocarboxylate transporter (MCT) family, which transport monocarboxylates such as lactate and pyruvate across peroxisomal membranes [82,83].

Like mitochondria, peroxisomes require shuttle systems to transfer reducing equivalents across the organellar membrane. Currently, there is some evidence that the redox equivalents of NADH, which are produced in the organelle lumen during peroxisomal β-oxidation (see Section 4.1), are transferred across the peroxisomal membrane via pyruvate/lactate- and dihydroxyacetone phosphate/glycerol-3-phosphate-based redox shuttles [78,82,84,85]. However, in contrast to what is known about the identity of the carriers involved in the transfer of redox equivalents across the IMM [86], it is not yet clear whether pyruvate, lactate, dihydroxyacetone phosphate, and glycerol-3-phosphate enter or escape peroxisomes predominantly through non-selective pores (e.g., PXMP2) or specific transporters (e.g., MCTs in case of lactate and pyruvate). A related question is how glutathione, ascorbic acid, and H_2_O_2_ (see Section 4.2.1) can penetrate the peroxisomal membrane. In this context, it is interesting to note that until now, and unlike in the IMM [87], no H_2_O_2_-permeable aquaporins have been identified in the peroxisomal membrane.

## 4. Metabolic Interplay

Over the past decades, it has become increasingly evident that diverse metabolic processes depend on the concerted action of both peroxisomes and mitochondria. Specific examples in mammals include β-oxidation of fatty acids, phytanic acid α-oxidation, bile acid synthesis, glyoxylate detoxification, and maintenance of cellular ROS homeostasis. For a detailed overview of the specific contributions of each organelle in any of these processes, we refer the reader to another recent review [88]. However, to illustrate the concept of metabolic cooperation between peroxisomes and mitochondria, we cover two examples in more detail in the following sections.

### 4.1. β-Oxidation of Fatty Acids

**Figure 3:**
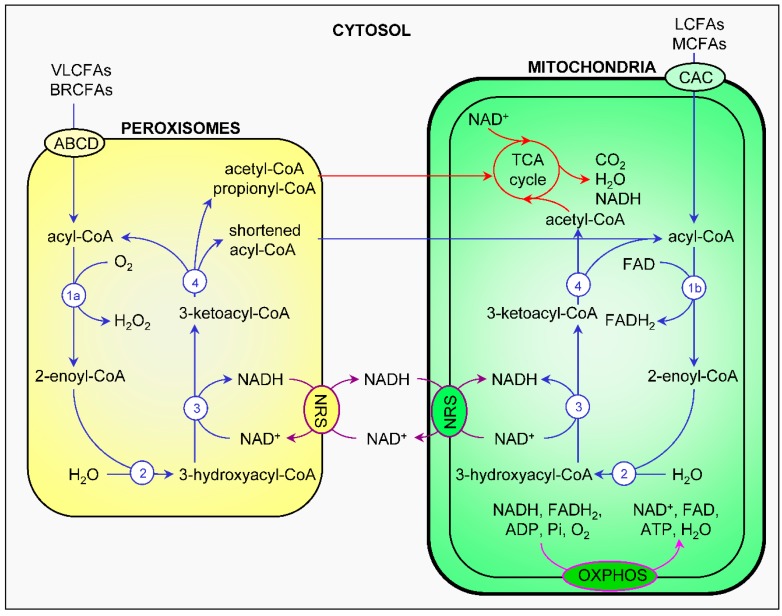
Comparison and interplay of peroxisomal and mitochondrial fatty acid β-oxidation (for details, see Section 4.1). Fatty acid β-oxidation, the NAD(H) redox shuttles, the tricarboxylic acid cycle, and the electron transfer chain are respectively depicted in blue, purple, red, and pink. 1a, acyl-CoA oxidase; 1b, acyl-CoA dehydrogenase; 2, enoyl-CoA hydratase; 3, 3-hydroxyacyl-CoA dehydrogenase; 4, 3-ketoacyl-CoA thiolase. ABCD, ATP-binding cassette transporters of subfamily D; ADP, adenine dinucleotide phosphate; BRCFA, branched-chain fatty acid; CAC, carnitine-acylcarnitine carrier; FAD, flavin adenine dinucleotide; FADH_2_, reduced FAD; LCFA, long-chain fatty acid; MCFA, medium-chain fatty acid; NAD, nicotinamide adenine dinucleotide; NADH, reduced NAD; NRS, NAD(H) redox shuttles; OXPHOS, oxidative phosphorylation; TCA, tricarboxylic acid; VLCFA, very-long-chain fatty acid.

Fatty acid β-oxidation is a multistep process by which fatty acyl-CoA esters are stepwise shortened between carbons 2 and 3, yielding as products: a chain-shortened acyl-CoA and—depending on the presence of a 2-methyl group in the substrate—acetyl-CoA or propionyl-CoA [89]. In mammals, this process takes place in both peroxisomes and mitochondria (Figure 3). Each β-oxidation cycle involves four consecutive reactions: (i) desaturation of the bond between C2 and C3; (ii) hydration of the formed 2-enoyl-CoA; (iii) dehydrogenation of 3-hydroxyacyl-CoA; and (iv) thiolytic cleavage of 3-oxoacyl-CoA [7]. While the three latter reactions are mechanistically comparable in both organelles, the first reaction is catalyzed by FAD-dependent acyl-CoA oxidases (ACOXs) in peroxisomes and FAD-dependent acyl-CoA dehydrogenases (ACADs) in mitochondria [26]. In the ACOX-catalyzed reaction, electrons from reduced FAD (FADH_2_) are passed directly to molecular oxygen (O_2_), thereby producing heat and H_2_O_2_ [26,88]; in the ACAD-catalyzed reaction, the electrons from FADH_2_ are delivered to the respiratory chain via the electron transfer flavoprotein (ETF) and the ETF dehydrogenase (ETFDH) [90].

Peroxisomes and mitochondria contain distinct sets of enzymes for each β-oxidation reaction step, and as such it is no surprise that both organelles have largely different substrate specificities [26,88]. For example, dietary fatty acids such as palmitic acid, oleic acid, and linoleic acid are preferentially β-oxidized in mitochondria [91]; and the broad range of carboxylates that cannot be β-oxidized by mitochondria (e.g., very-long-chain fatty acids (VLCFAs), pristanic acid and other 2-methyl-branched prostanoids, and bile acid intermediates) are substrates for peroxisomal β-oxidation [7]. In addition, the end products of peroxisomal and mitochondrial β-oxidation are different: while peroxisomes are only able to chain-shorten their substrates, mitochondria can β-oxidize fatty acids all the way to CO_2_ and H_2_O through entry of acetyl-CoA into the tricarboxylic acid cycle (TCA) and reoxidation of NADH and FADH_2_ by the respiratory chain [88]. Importantly, the latter process can generate 2 ATP molecules per cleavage cycle. The peroxisomal β-oxidation products can only be fully oxidized to CO_2_ and H_2_O after they have been shuttled to mitochondria. In order to keep peroxisomal β-oxidation ongoing, it is crucial that NADH is reoxidized to NAD^+^ [89]. This process is thought to rely on a redox shuttle mechanism that involves mitochondria (see Section 3.3).

### 4.2. Reactive Oxygen Species (ROS) Metabolism

Emerging evidence suggests that peroxisomes and mitochondria in mammals harbor sophisticated redox control mechanisms that integrate environmental cues and control various physiological and metabolic functions [92]. In the following sections, we briefly review their main pro- and antioxidant capacities and what is known about their redox-governed relationship. For a more detailed description of the peroxisomal and mitochondrial pro- an antioxidant systems, we refer the reader to another review [92].

#### 4.2.1. ROS/RNS Producing and Scavenging Enzymes

In general, mitochondria are considered to be a primary source of ROS within cells. Conventionally, the respiratory complexes I and III are considered the major contributors to mitochondrial ROS production [93]. However, there are also many other sites within mitochondria where ROS/RNS can be formed [94,95,96]. The primary type of ROS released by complex I and complex III is O_2_^•−^, which is rapidly converted to H_2_O_2_ by SOD1 (when the radical is produced in the intermembrane space) or SOD2 (when the radical is produced inside the mitochondrial matrix) [97]. Besides SOD1 and SOD2, mitochondria also contain other antioxidant defense systems, including components of the glutathione/glutaredoxin and thioredoxin/peroxiredoxin systems [93] and low molecular weight antioxidants such as CoA [98,99], ubiquinol [100], vitamin C [101], and vitamin E [102]. The amount of ROS released from mitochondria appears to depend on the physiological state and growth environment of the cell [103,104,105].

As befits their name, peroxisomes play an important role in cellular H_2_O_2_ metabolism. This is perhaps best demonstrated by the fact that they contain multiple FAD-containing oxidoreductases that produce H_2_O_2_ as part of their normal catalytic activity (see Section 4.1), as well as catalase, one of the best characterized H_2_O_2_-degrading enzymes [106]. Depending on the organism, tissue, and cell type, mammalian peroxisomes may also contain xanthine oxidoreductase (XDH) and inducible nitric oxide synthase (NOS2), two potential sources of superoxide (O_2_^•−^) and nitric oxide (^•^NO) [92]. To prevent the accumulation of ROS/RNS and oxidative damage, the organelles possess, besides catalase, other enzymatic (e.g., peroxiredoxin-5 (PRDX5) and superoxide dismutase 1 (SOD1)) and non-enzymatic (e.g., ascorbic acid and glutathione) antioxidant systems [5,6]. Currently, it is not clear how these low molecular weight compounds are transported across the peroxisomal membrane and whether or not mammalian peroxisomes contain a functional glutathione redox system (see Section 3.3). Another unresolved question in the field is whether peroxisomes act as a net source or sink for ROS/RNS. However, as is the case for mitochondria, it is very likely that this will depend on the physiological state and growth environment of the cell [92].

#### 4.2.2. Peroxisome-Mitochondria Redox Interplay

Currently, there is ample evidence that, at least in mammals, peroxisomes and mitochondria share a redox-sensitive relationship. For example, defects in catalase activity [107,108,109,110], peroxisomal β-oxidation [111,112], or peroxisome biogenesis [57,58,62,113] have been shown to induce mitochondrial oxidative stress in various organs (e.g., liver [57,113], proximal tubules of the kidney [109,113], adrenal cortex [113], spinal cord [111,112], heart [113], and brain [62]) and cell types (e.g., fibroblasts [107,108,110,111], mesanglial cells [109], skeletal and smooth muscle cells [58], and hepatocytes [57]). Interestingly, this increase in mitochondrial redox state closely follows the induction of peroxisomal function loss [57,108,110]. In the case where peroxisome biogenesis is completely blocked, such treatment also results in other mitochondrial perturbations, including structural alterations of the inner mitochondrial membrane [57,58,59], a reduction in the activities of multiple respiratory chain complexes [57,58,59], reduced mitochondrial DNA abundance [57], and an increase in mitochondrial volume [57,59]. On the other hand, an increase in catalase activity [109,114], peroxisomal β-oxidation [115] or peroxisome number [116] has been reported to ameliorate mitochondrial fitness and protect these organelles against oxidative insults. In reverse, to the best of our knowledge, there are no published studies that have addressed how specific defects in mitochondrial functions affect the redox state of peroxisomes, and this issue remains an unresolved open question.

## 5. Interorganelle Signaling

From the previous sections, it is clear that peroxisomal and mitochondrial metabolic activities are closely intertwined, bolstering the idea that these organelles have developed strategies to communicate their fitness levels to each other. Currently, little is known about the mechanisms and messengers that convey this information from one organelle to the other. However, in analogy with other mitochondrial signaling pathways [15], potential communication strategies may include (i) the release of biomolecules (e.g., proteins, ROS/RNS, or metabolites) that directly or indirectly (e.g., through epigenetic modifications or retrograde signaling pathways) modulate the other organelle’s function; or (ii) the activation of membrane-associated signaling scaffolds that coordinate the activity of both organelles.

### 5.1. Release of Messenger Molecules

#### 5.1.1. ROS

As highlighted in Section 4.2.1, peroxisomes and mitochondria play a central role in the metabolism of H_2_O_2_, a key signaling molecule in cellular redox signaling [117]. In general, it is thought that H_2_O_2_ functions as a signaling molecule through oxidation of deprotonated (low pKa) protein cysteine residues that are highly conserved in various signaling proteins, including some transcriptional regulators, kinases, phosphatases, metabolic enzymes, structural proteins, and proteases [118]. This will lead to the formation of unstable sulfenic acid intermediates that are subsequently reduced to a disulfide bond by reaction with glutathione or inter- or intramolecular protein thiols [119]. As reversible disulfide bond formation often serves as an “on” or “off” switch that regulates protein activity, localization, and/or interaction with other biomolecules [120,121], changes in H_2_O_2_ flux can be expected to affect diverse cellular processes.

Given that H_2_O_2_ can rapidly cross the peroxisomal [122] and mitochondrial [87] membrane (see also Section 3.3), it is reasonable to expect that alterations in peroxisomal or mitochondrial H_2_O_2_ metabolism will also impact the function of the other organelle. The observation that changes in peroxisomal catalase activity affect mitochondrial redox state and function is in line with this idea [107,108,110,114,123,124]. However, at the moment, it remains to be determined whether peroxisomal H_2_O_2_ acts directly or indirectly (e.g., through oxidation of H_2_O_2_ scavenging enzymes that subsequently transfer the oxidative equivalents to other target proteins via thiol-disulfide interchange reactions [125,126] and/or by activation of stress response pathways) on mitochondria [92]. In this context, it is worth noting that inhibition of peroxisomal catalase activity not only rapidly increases mitochondrial oxidative damage [110], but also results in reduced expression of peroxisome proliferator-activated receptor (PPAR)-γ co-activator (PGC) 1α, a master regulator of mitochondrial biogenesis and function [123]. Once again, little is known about whether or not changes in mitochondrial H_2_O*_2_* metabolism also affect peroxisomal redox state. However, there is good evidence that moderate levels of mitochondrial ROS can promote the expression of stress-responsive transcription factors (e.g., nuclear respiratory factor (NRF) 2 and Forkhead box O (FOXO)) that mediate stress tolerance through upregulation of multiple antioxidant enzymes, including catalase [127,128,129]. These findings are in line with a study in *Saccharomyces cerevisiae* showing that respiratory deficiency, but not inhibition of mitochondrial ATP synthesis per se, dramatically induces peroxisome biogenesis and function through activation of retrograde signaling pathways [130].

Finally, targeted variants of KillerRed, a red fluorescent photosensitizer that efficiently generates O_2_^•−^ upon green light illumination [131], have been employed to study redox communication between peroxisomes and mitochondria in mammalian cells [108,132,133]. On one hand, these studies confirmed and extended the concept that mitochondrial redox balance is quickly perturbed upon generation of excess ROS inside peroxisomes. On the other hand, they provided the first evidence that (i) peroxisomes largely resist oxidative stress when the burden originates within mitochondria [108]; and (ii) redox communication between peroxisomes and mitochondria involves complex signaling pathways [132,133]. Importantly, although the identity and mechanisms of these pathways remain to be elucidated (and may differ according to the type and amount of ROS produced), the data presented in this section strongly support the idea that mitochondria may act as dynamic receivers, integrators, and transmitters of peroxisome-derived mediators of oxidative stress [132].

#### 5.1.2. Lipids

Given that peroxisomes and mitochondria play a central role in cellular lipid metabolism [92], and that lipids play multiple roles in cellular signaling, bioenergetics, and membrane structure and function [134], changes in peroxisomal or mitochondrial lipid metabolism would be expected to influence the function of the other organelle. In the following paragraphs, we provide evidence that this is indeed the case.

Specific or general defects in peroxisome function can result in the accumulation of phytanic acid (e.g., in patients with α-oxidation defects), pristanic acid (e.g., in patients with peroxisomal β-oxidation defects) and/or VLCFAs (e.g., in X-linked adrenoleukodystrophy (X-ALD) or Zellweger syndrome spectrum (ZSS) patients) [8], and most—if not all—of these defects have been directly or indirectly linked to mitochondrial dysfunction [92]. For example, treatment of various cell types (e.g., rat pheochromocytoma cells [103], astrocytes [135,136] or oligodendrocytes [136,137] from mouse [137] or rat [103,135,136]) or mitochondrial fractions derived from rat brain [135,138] with any of these carboxylates has been shown to induce mitochondrial depolarization [135,136], respiratory chain dysfunction [135,138], oxidative stress [103,135,137], vacuolization [138], and/or the release of cytochrome c [135] in association with cell death [135,136]. It is important to mention that all these experiments have been carried out with supra-physiological fatty acid concentrations and, at physiological levels, no such abnormalities could be observed, at least not in wild-type cells [137]. Nevertheless, these findings strongly indicate that elevated levels of peroxisomal fatty acid oxidation substrates can induce multifaceted deficits in mitochondria.

To the best of our knowledge, there are few studies that directly examine how defects in mitochondrial fatty acid oxidation influence peroxisome physiology. One study has demonstrated that pharmacological inhibition of mitochondrial β-oxidation in human and rat liver slices is associated with a time- and inhibitor concentration-dependent upregulation of peroxisomal β-oxidation gene transcripts [60]. Another study on skeletal muscle has shown that impaired mitochondrial fatty acid oxidation leads to a compensatory increase in peroxisomal fatty acid oxidation [61].

#### 5.1.3. Other Metabolites

Multiple peroxisomal (e.g., fatty acid α- and β-oxidation) and mitochondrial (e.g., fatty acid β-oxidation, TCA cycle, and respiratory chain) processes involve common co-substrates (e.g., FAD, NAD^+^, O_2_, and α-ketoglutarate) and metabolites (e.g., acetyl-CoA, succinate) that have the potential to directly or indirectly modulate the metabolic activities of the other subcellular compartment. For example, peroxisomal β-oxidation can only continue if the NADH formed in peroxisomes is reoxidized to NAD^+^, a complex process that can only be achieved in mitochondria (see Section 3.3) [88]. In addition, as many of these co-substrates and metabolites can also serve as substrates or inhibitors of DNA methyltransferases, histone (de)methyltransferases, and histone (de)acetylases, changes in peroxisomal or mitochondrial activity are likely to influence the activity of the other organelle through epigenetic remodeling [139,140].

#### 5.1.4. Proteins

More than 20 years ago, it was reported that mitochondria can communicate with the cell through release of cytochrome c, a central event in apoptotic signaling [141]. In the meantime, it is well-known that also other death-promoting factors residing in the mitochondrial inner membrane space can be released into the cytosol upon induction of apoptosis [142]. However, only very recently it has been shown that peroxisomes can also release matrix proteins into the cytosol [143]. Interestingly, this process appears to depend on voltage-dependent anion-selective channel (VDAC) 2 [143], a redox-sensitive outer mitochondrial membrane (OMM) protein whose primary role is to form an aqueous pore allowing the exchange of small ions and metabolites across the OMM [144]. Loss of VDAC2 in Chinese hamster ovary cells shifts the localization of BCL2-antagonist/killer (BAK) 1, a B-cell lymphoma (BCL) 2 family member that is at the core of the mitochondrial pathway of apoptosis [145], from mitochondria to peroxisomes, thereby increasing the permeability of the peroxisomal membrane in a manner akin to OMM permeabilization [143]. This in turn results in the release of peroxisomal matrix proteins, including catalase, into the cytosol. Although we and others have demonstrated that mislocalization of catalase to the cytosol provides protection against externally added H_2_O_2_ [143,146], the physiological relevance of the VDAC2 observations remains to be established.

### 5.2. Membrane-Associated Signaling Scaffolds

In the last decade, emerging evidence has highlighted the central role of membrane-bound signaling complexes in diverse cellular processes [147,148]. One such signaling complex, containing mitochondrial antiviral-signaling protein (MAVS), has been shown to reside on mitochondria [149], peroxisomes [150], and MAMs [65]. MAVS is a crucial adaptor protein that binds activated RIG-I-like receptors (RLRs), which are pathogen recognition receptors that detect foreign cytosolic RNA species [151]. Upon binding to MAVS, RLRs induce a series of signaling events eventually leading to the production of pro-inflammatory cytokines and interferons (IFNs) of the type I and III families, thereby providing the first line of cellular defense against pathogen invasion [151]. Activation of peroxisomal and mitochondrial MAVS has been shown to induce type III and type I IFN expression, respectively [152]. The same study also demonstrated that the strength of the type III IFN response can be directly linked to peroxisome abundance. Others have demonstrated that cells expressing MAVS exclusively on peroxisomes can also induce type I IFN expression upon infection with diverse RNA viruses [153]. The precise reasons for these apparently conflicting findings are not clear. Nevertheless, these studies clearly demonstrate the cooperative role of peroxisomes and mitochondria, thereby allowing the cell to mediate its antiviral activities according to the type of virus and stage of infection [65,150,152,153,154].

## 6. Peroxisome-Mitochondria Communication: Physiological Importance in Health and Disease

Peroxisomes and mitochondria cooperate in multiple metabolic and signaling networks (see Section 4 and Section 5, respectively) and their abundance is co-regulated at different levels (see Section 2). In the following sections, we outline what is currently known about the interplay between these organelles under various disease conditions. Unfortunately, relatively little information is available on how peroxisome biology is affected in cells from patients suffering from mitochondrial disease. One major reason for this is that, until recently, peroxisomal parameters were not documented because the organelles were often dismissed as the cellular hoi polloi [29]. Given that functional impairment of either peroxisomes or mitochondria is likely to induce dysfunction of the other (see above), the precise contribution of each organelle to disease pathology and development is not yet clear and likely to be complex.

### 6.1. Organelle Function Deficiencies

Mutations in proteins impairing peroxisome or mitochondrial biogenesis and/or function have been shown to lead to inherited disorders with different, and severe, phenotypic presentations [8,155]. To gain a better insight into the mechanisms of disease pathogenesis, multiple mouse models have been generated [156,157,158,159,160,161]. From these and other studies, it has become increasingly clear that defects in peroxisome biogenesis (e.g., in ZSS patients) [58], peroxisomal fatty acid metabolism (e.g., in X-ALD patients) [112], or peroxisomal antioxidant capacity (e.g., in acatalasemia patients) [162] have a negative impact on mitochondrial functioning. In general, such defects have been reported to induce ultrastructural and/or functional mitochondrial alterations such as abnormal cristae, a decrease in membrane potential and respiration rates, increased ROS production, reduced fatty acid oxidation, DNA depletion, and/or an increase in mass in various organs (e.g., brain, liver, and kidney) and cell types (e.g., skeletal and smooth muscle cells) [62,111,113,163,164,165]. The molecular mechanisms underlying these mitochondrial changes remain poorly understood. However, given that peroxisomes play a central role in cellular lipid and ROS metabolism, mitochondrial dysfunction is likely to be caused by changes in the lipid composition of their membranes and the buildup of cellular ROS [92]. In this context, it is interesting to note that inactivation of peroxisomal acyl-CoA oxidase 1 (ACOX1) in mice, a condition associated with microvesicular steatohepatitis, has been reported to induce mitochondrial damage through (i) a sustainable activation of PPARα by natural ligands that remain unmetabolized in the absence of ACOX1; (ii) the PPARα-induced expression of cytochrome P450 CYP4A ω-oxidation enzymes, which metabolize long-chain fatty acids (LCFAs) to dicarboxylic acids; and (iii) the accumulation of dicarboxylic acids, which are normally degraded by peroxisomal β-oxidation, to concentrations that are sufficient to uncouple oxidative phosphorylation ([160,161,166], and references therein). Another pertinent question is how mitochondrial dysfunction contributes to the clinical phenotype of a heterogeneous group of patients with primary defects in peroxisome function. In this context, it is interesting to note that a recent study has shown that patients with Zellweger syndrome, the prototype of neonatal peroxisomal disease, present with clinical features of mitochondrial myopathy, thereby underscoring the role of secondary mitochondrial dysfunction in the disease phenotype [58]. Whether or not secondary dysfunction of peroxisomes also contributes to the disease phenotype of patients with primary mitochondrial defects, remains to be investigated.

### 6.2. Defects in Shared Components of the Fission Machinery

Defects in shared components of the peroxisomal and mitochondria fission machinery have been found to underlie several human degenerative disorders. For example, defects in DNM1L have been associated with the autosomal dominant disorder encephalopathy due to defective mitochondrial and peroxisomal fission (EMPF), a lethal disorder characterized by cerebral dysgenesis, optic atrophy and hypoplasia, and seizures [167]; infantile encephalopathy [168]; and refractory epilepsy [169]. Loss of MFF has been linked to mitochondrial encephalomyopathy [170] and early-onset Leigh-like encephalopathy, optic atrophy, and peripheral neuropathy [171]; and mutations in GDAP1 have been shown to cause Charcot-Marie-Tooth disease, the most common inherited peripheral neuropathy [172]. Interestingly, in contrast to the dual peroxisomal and mitochondrial fission defect observed in skin fibroblasts from patients with DNM1L or MFF mutations [167,168,169,170,171], the currently identified missense disease mutants of GDAP1 appear to impair only mitochondrial fragmentation in mouse neuroblastoma N1E-115 cells (loss of GDAP1 also leads to peroxisomal elongation) [47]. Whether or not such GDAP1 mutations affect other peroxisomal parameters, remains to be determined. In this context, it is interesting to point out that the EMPF patient presented with combined features of mitochondrial (e.g., autosomal dominant optic atrophy, neuropathy, and lactic acidosis) and peroxisomal (e.g., dysmyelination and mildly elevated levels of VLCFAs) disorders [167].

### 6.3. Dysregulation of Pexophagy

Growing evidence suggests that changes in peroxisome turnover rates can affect human physiology and pathology [116,173,174,175]. Interestingly, one of these studies found that severe malnutrition in young children, a condition associated with signs of hepatic dysfunction such as steatosis and hypoalbuminemia, resulted in an almost complete disappearance of hepatic peroxisomes [173]. Recently, this finding was confirmed and extended by others, who developed a rat model of malnutrition and demonstrated that (i) prolonged dietary protein restriction decreases mitochondrial fitness in hepatocytes from weanling rats; (ii) this decrease in mitochondrial fitness is caused by enhanced peroxisome degradation; and (iii) fenofibrate supplementation, a condition enhancing hepatic peroxisome abundance (see Section 2.1), recovers hepatic mitochondrial function, thereby reducing steatosis and restoring plasma albumin levels [116]. Taken together, these studies once again underline the importance of functional peroxisomes for maintenance of mitochondrial fitness.

### 6.4. Viral Infections

In recent years, it has become increasingly clear that peroxisomes and mitochondria cooperate to combat viral infections through activation of the RLR-MAVS signaling pathway (see Section 5.2). In addition, it has been shown that (i) several RNA viruses that are of great importance for global public health (e.g., rotavirus, HIV, and influenza) exploit these organelles in their replication cycle [176,177,178]; (ii) pathogenic flaviviruses such as West Nile and dengue impair peroxisome biogenesis in infected cells, thereby suppressing the induction of type III IFNs [179]; and (iii) hepatitis B virus X protein, a multifunctional viral protein that binds to MAVS and promotes the development and progression of hepatocellular carcinoma, triggers the production of ROS and activation of NF-κB upon association with mitochondria [180] or peroxisomes [181]. In summary, these observations highlight the critical and cooperative role of peroxisomes and mitochondria in antiviral defense. A major challenge in the future will be to translate these in cellulo findings into clinically relevant in vivo models.

### 6.5. Cellular Aging and Age-Related Diseases

Currently, there is compelling evidence to suggest that both peroxisomal and mitochondrial dysfunction can contribute to organismal aging and age-related diseases [182,183]. However, at the moment, little is known about how disturbances in the bidirectional communication axes between peroxisomes and mitochondria influence age-related disease development. Nevertheless, in analogy with what has been observed in cells from patients suffering from congenital peroxisomal disorders (see Section 6.1) or rats subjected to prolonged dietary protein restriction (see Section 6.3), it is plausible to assume that peroxisomes also serve as guardians of mitochondrial fitness during cellular aging and age-related disease development. In line with this view, it has been demonstrated that (i) restoration of peroxisomal catalase import in a late-passage (Hs27) human diploid fibroblasts reverses mitochondrial depolarization and delays the appearance of the senescence-associated marker β-galactosidase [114]; and (ii) peroxisome proliferation as well as increased peroxisomal antioxidant activity can protect hippocampal neurons from amyloid-β peptide-induced mitochondrial dysfunction, neurotoxicity, and cell death [184,185].

## 7. Conclusions, Challenges and Perspectives

As reviewed above, an overwhelming body of evidence indicates that diverse cellular metabolic and signaling processes depend on the concerted action of both peroxisomes and mitochondria. Examples have been presented to support the view that peroxisomal and mitochondrial abundance and enzyme content are controlled by common sets of *cis*- and *trans*-acting factors. In addition, it is clear that both organelles have the ability to convey information from one to the other through the release of biological messengers such as ROS, lipids, and other metabolites. However, the molecular details of many of these processes and events remain unclear. For example, almost nothing is known about if and how mitochondrial defects affect peroxisome function; little is known about how peroxisomes and mitochondria exchange co-factors, metabolites, and signaling molecules; and it remains to be established how peroxisome deficits induce mitochondrial dysfunction. A major challenge is to translate the in cellulo findings of most studies into physiological and clinically relevant in vivo models. However, given that the contribution of each organelle to disease pathology and development is likely multifaceted and complex, this will not be an easy task. Nevertheless, gaining more insight into the intricate relationship between peroxisomes and mitochondria will provide important clues to understanding the role of both organelles in health and disease.

## Abbreviations

ABCD	ATP-binding cassette transporters of subfamily D
ACAD	Acyl-CoA dehydrogenase
ACOX	Acyl-CoA oxidase
ADP	Adenine dinucleotide phosphate
ATP	Adenosine triphosphate
BAK	BCL2-antagonist/killer
BCL	B-cell lymphoma
BRCFA	Branched-chain fatty acid
CAC	Carnitine-acylcarnitine carrier
cAMP	Cyclic adenosine monophosphate
CoA	Coenzyme A
DNM1L	Dynamin 1-like protein
ECI	Enoyl-CoA δ isomerase
EMPF	Encephalopathy due to defective mitochondrial and peroxisomal fission
ER	Endoplasmic reticulum
ETF	Electron transfer flavoprotein
ETFDH	ETF dehydrogenase
FA	Fatty acid
FAD	Flavin adenine dinucleotide
FADH_2_	Reduced FAD
FIS	Mitochondrial fission protein
FOXO	Forkhead box O
GDAP	Ganglioside-induced differentiation-associated protein
IFN	Interferon
IMM	Inner mitochondrial membrane
LCFA	Long-chain fatty acid
MAM	Mitochondria-associated ER membrane
MAVS	Mitochondrial antiviral-signaling protein
MCFA	Medium-chain fatty acid
MCT	Monocarboxylate transporter
MDV	Mitochondria-derived vesicle
MIEF	Mitochondrial elongation factor
MFF	Mitochondrial fission factor
MT	Mitochondria/mitochondrial
MUL	Mitochondrial E3 ubiquitin protein ligase
NAD^+^	Nicotinamide adenine dinucleotide
NADH	Reduced NAD^+^
NCOR	Nuclear receptor corepressor
NOS	Nitric oxide synthase
NRF	Nuclear respiratory factor
NRS	NAD(H) redox shuttle
OMM	Outer mitochondrial membrane
OXPHOS	Oxidative phosphorylation
PEX	peroxin
PGC	PPAR-γ co-activator
PO	Peroxisome/peroxisomal
PPAR	Peroxisome proliferator-activated receptor
PRDX	Peroxiredoxin
PXMP2	Peroxisomal membrane protein 2
RIG-I	Retinoic acid-inducible gene 1
RLR	RIG-I-like receptor
RNS	Reactive nitrogen species
ROS	Reactive oxygen species
RXR	Retinoid X receptor
SOD	Superoxide dismutase
TCA	Tricarboxylic acid
TF	Transcription factor
VDAC	Voltage-dependent anion-selective channel
VLCFA	Very-long-chain fatty acid
X-ALD	X-linked adrenoleukodystrophy
XDH	Xanthine oxidoreductase
ZSS	Zellweger syndrome spectrum
